# Improved health-related quality of life in patients treated with topical sirolimus for facial angiofibroma associated with tuberous sclerosis complex

**DOI:** 10.1186/s13023-020-01417-5

**Published:** 2020-06-01

**Authors:** Takashi Hatano, Yuki Ohno, Yu Imai, Jun Moritake, Katsuhisa Endo, Mayumi Tamari, Shin Egawa

**Affiliations:** 1 Tuberous Sclerosis Complex Unit, TSC unit, JR Tokyo General Hospital, 2-1-3 Yoyogi Shibuya-ku, Tokyo, 151-8528 Japan; 2 Department of Dermatology, JR Tokyo General Hospital, Tokyo, Japan; 3 Department of Urology, JR Tokyo General Hospital, Tokyo, Japan; 4 Research Center for Medical Science, Division of Molecular Genetics, The Jikei University School of Medicine, Tokyo, Japan; 5 Department of Urology, The Jikei University School of Medicine, Tokyo, Japan

**Keywords:** Tuberous sclerosis complex, Facial angiofibroma, Topical sirolimus, Health-related quality of life, Mental health

## Abstract

**Background:**

Tuberous sclerosis complex (TSC) is a rare autosomal dominant disorder forming hamartomas throughout the body. Facial angiofibromas (FAs) occur in 75% of TSC patients, which are often enlarged, impairing the appearance of the face, and reducing the patient’s quality of life (QOL). The aim of this study was to characterize the impact of topical sirolimus treatment on the health-related QOL in patients with FA associated with TSC.

**Methods:**

We investigated a total of 33 patients who received sirolimus gel treatment for FA associated with TSC and assessed the changes in the health-related QOL using the Medical Outcomes Study 36-Item Short Form (SF-36) Health Survey. SF-36 surveys were performed before and after 3 months of treatment. The conditions of the patients after using the sirolimus gel were categorized into the following three categories: “improved,” “unchanged,” and “aggravated.” Adverse events were investigated using the CTCAE v5.0-JCOG.

**Results:**

The median age of the patients was 25 (range 14–55) years. After 3 months of sirolimus gel treatment, three scale scores of the SF-36, vitality (VT), social function (SF), and mental health (MH), were significantly improved compared to before the treatment. The VT and SF in patients who had improved FA were significantly better than those in the other patients. There were no significant differences in any scale scores between patients with and without adverse events at 3 months after the initiation of sirolimus gel treatment.

**Conclusions:**

This is the first report regarding improved health-related quality of life in patients treated with sirolimus gel for FA associated with TSC by using the SF-36. The three scale scores associated with mental health were significantly improved compared to before the treatment. The health-related QOL in patients receiving sirolimus gel treatment is more strongly affected by the treatment efficacy than adverse events.

**Mini-abstract:**

Sirolimus gel treatment improves the health-related QOL in patients with FA associated with TSC.

## Background

Tuberous sclerosis complex (TSC) is a rare autosomal dominant inherited disorder characterized by systemic hamartomas and hypomelanotic macules [1]. It is caused by genetic mutations of either the *TSC1* gene on chromosome 9 or the *TSC2* gene on chromosome 16, which encode hamartin and tuberin, respectively [2, 3]. Dysfunction of hamartin or tuberin induces the constitutive activation of mammalian target of rapamycin complex 1 (mTORC1) [4]. Most symptoms of TSC are considered to be derived from the activation of mTORC1. Since approximately 75% of patients with TSC suffer from facial angiofibromas (FAs), which are often enlarged, impairing the appearance of the face, and reducing the patient’s quality of life (QOL), patients with FAs need to be treated.

Wataya-Kaneda et al. reported phase 2 and 3 randomized clinical trials of topical sirolimus treatment with a sirolimus gel formulation in TSC patients and showed significant reductions in the size and color of the FAs associated with TSC [5, 6]. In Japan, sirolimus gel was approved for the treatment of TSC skin lesions in 2018. However, few studies that assess the effect of topical sirolimus treatment on the health-related QOL in patients with FA associated with TSC have been conducted.

In the present study, we evaluated the changes in the health-related QOL of patients receiving sirolimus gel treatment for FA associated with TSC, using the Medical Outcomes Study 36-Item Short Form (SF-36) Health Survey, one of the most widely used surveys for health-related QOL [7].

## Methods

### Study design and patients

FA associated with TSC was diagnosed based on the International Tuberous Sclerosis Complex Consensus Conference (ITSCCC) diagnostic criteria after consultations with an internist and dermatologist. A total of 33 patients met the diagnostic criteria and received topical sirolimus (Raparimus^Ⓡ^ gel; Nobelpharma, Tokyo, Japan). The sirolimus gel contained 0.2% sirolimus and additives including alcohol. Each patient was instructed to spread 400 mg of the sirolimus gel evenly on the FA site twice a day. The severity of FA was assessed using the Facial Angiofibroma Severity Index [8]. The conditions of the patients after 3 months of treatment with sirolimus gel were classified into three categories according to the criteria shown in Table 1: “improved,” “unchanged,” and “aggravated.” [6] Adverse events were investigated using the CTCAE v5.0-JCOG (National Cancer Institute, Bethesda, MD, USA). This study was approved by the institutional review board of JR Tokyo General Hospital (No. H29–27).

**Table 1:** The criteria for improvements in facial angiofibroma lesions from baseline

Rating	Size	Color
Improved	Reduced in ≥50% of lesions	Improved by ≥2 reddishness levels in ≥50% of lesions
Unchanged	Not obviously changed	Not obviously changed
Aggravated	Increased or newly formed papules in ≥50% of lesions	Aggravated by ≥2 reddishness levels in ≥50% of lesions

### Exclusion criteria

The following patients were excluded: those with erosions, ulcers, or other skin lesions associated with FAs, uncontrollable epileptic seizures despite being treated with anti-epileptic agents, poor respiratory condition due to lung lymphangioleiomyomatosis, or systemic treatment with an mTOR inhibitor; and those who were pregnant or who could not periodically visit the hospital. Since children were frequently unable to respond appropriately to the SF-36 questionnaire, we therefore excluded pediatric patients from the study.

### Assessment of the health-related QOL

For assessment of the health-related QOL in 33 patients, the Japanese version 2 of the SF-36 was used [7]. This questionnaire is completed by patient self-report through survey or interview, and consists of 36 self-administered questions that quantify the health-related QOL using 8 multi-item scales for the health status covering both the mental and physical aspects of health, namely the physical function (PF), role limitations because of physical health problems (RP), bodily pain (BP), general health perception (GH), vitality (VT), social function (SF), role limitations because of emotional problems (RE), and mental health (MH). The PF, RP, BP, GH and VT indicate physical health, and the GH, VT, SF, RE and MH indicate mental health. Each domain is scored on a scale of 0 to 100, with lower scores indicating a poorer health status. We standardized these domain scores using Japanese population norms to give mean scores of 50 and standard deviations of 10 [7, 9]. In this study, SF-36 surveys were performed before and at 3 months after the initiation of sirolimus gel treatment.

### Statistical analyses

The SF-36 scores are presented as the mean and the standard deviation. The pre- and post-treatment scores were analyzed using unpaired t-tests. A *P* level of < 0.05 was considered to be statistically significant.

## Results

### Patient characteristics, treatment efficacy and adverse events

We analyzed 33 patients with FA associated with TSC who received sirolimus gel treatment (Table 2). The median age of the patients was 25 (range 14–55) years. The performance status was 2 in one patient who showed muscle weakness. Sirolimus gel treatment improved FA associated with TSC in 23 of the 33 (70%) patients after 3 months of treatment. However, the conditions of the remaining 10 (30%) were unchanged. None of the conditions of these patients were classified as “aggravated.” The severity of FA at the initiation of treatment did not differ between the improved group and the unchanged group. Adverse events related to sirolimus gel were observed in 12 (36%) patients. The major adverse events were acne, application site irritation, dry skin, and pruritus. There were no cases of grade ≥ 3 adverse events. Table 3 compares the treatment-related adverse events of the improved group and the unchanged group. All patients’ events improved with symptomatic treatment. None of the patients discontinued the treatment due to adverse events.

**Table 2:** Baseline characteristics of 33 patients receiving sirolimus gel for facial angiofibroma associated with tuberous sclerosis complex

Characteristics	Number	%
Median age (range)	25 (14–55)	
Sex		
Male/Female	17/16	
ECOG performance status		
0/1/2	24/8/1	
**Major features**		
≥ 3 Hypomelanotic macules	20	61
≥ 2 Ungual fibromas	18	55
Shagreen patch	17	52
Multiple retinal hamartomas	6	18
Cortical dysplasias	25	76
Subependymal nodules	19	58
Subependymal giant cell astrocytoma	5	15
Cardiac rhabdomyoma	4	12
Lung lymphangioleiomyomatosis	6	18
≥ 2 Renal angiomyolipomas	31	94
**Minor features**		
≥ 3 Dental enamel pits	10	30
≥ 2 Intraoral fibromas	6	18
Retinal achromic patch	5	15
Multiple renal cysts	2	6
Nonrenal hamartomas	1	3

**Table 3:** The incidence rates of treatment-related adverse events

Event	Improved group (*n* = 23)	Unchanged group (*n* = 10)
Acne	2 (9%)	2 (20%)
Application site irritation	2 (9%)	1 (10%)
Dry skin	3 (13%)	0 (0%)
Pruritus	1 (4%)	1 (10%)

### Evaluation of the health-related QOL

**Fig. 1:**
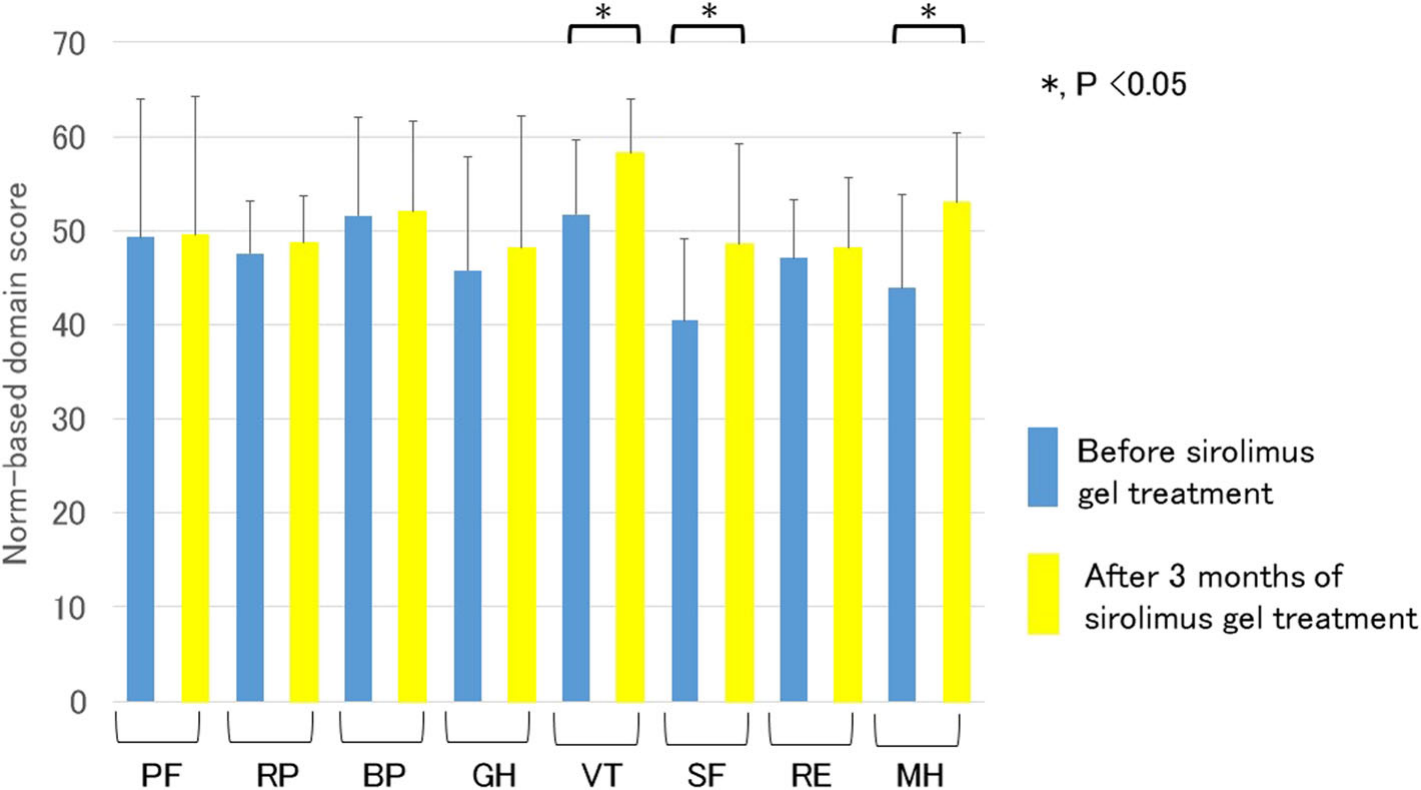
Comparison of the scale scores on the Medical Outcomes Study 36-Item Short Form in patients with sirolimus gel treatment for facial angiofibroma with tuberous sclerosis complex before and after 3 months of treatment. PF, physical function; RP, role limitations because of physical health problems; BP, bodily pain; GH, general health perception; VT, vitality; SF, social function; RE, role limitations because of emotional problems; MH, mental health. *, *P* < 0.05. The scale scores of the VT, SF, and MH were significantly improved compared to before the treatment

We evaluated the changes in the health-related QOL in patients with sirolimus gel treatment for FA associated with TSC by using the SF-36. After 3 months of sirolimus gel treatment, three scale scores of the SF-36 (VT, SF, and MH) were significantly improved compared to before treatment (Fig. 1). For the remaining five items, there were no marked differences after treatment compared with before treatment.

**Fig. 2:**
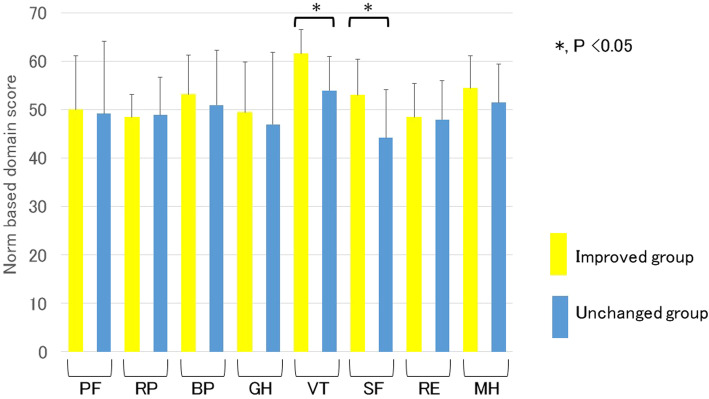
Comparison of the scale scores on the Medical Outcomes Study 36-Item Short Form 3 months after the initiation of sirolimus gel treatment for facial angiofibroma associated with tuberous sclerosis complex between patients with and without improved facial angiofibroma. PF, physical function; RP, role limitations because of physical health problems; BP, bodily pain; GH, general health perception; VT, vitality; SF, social function; RE, role limitations because of emotional problems; MH, mental health. *, *P* < 0.05. The scale scores of the VT and SF in patients whose FA had improved were significantly higher than those whose condition did not improve

**Fig. 3:**
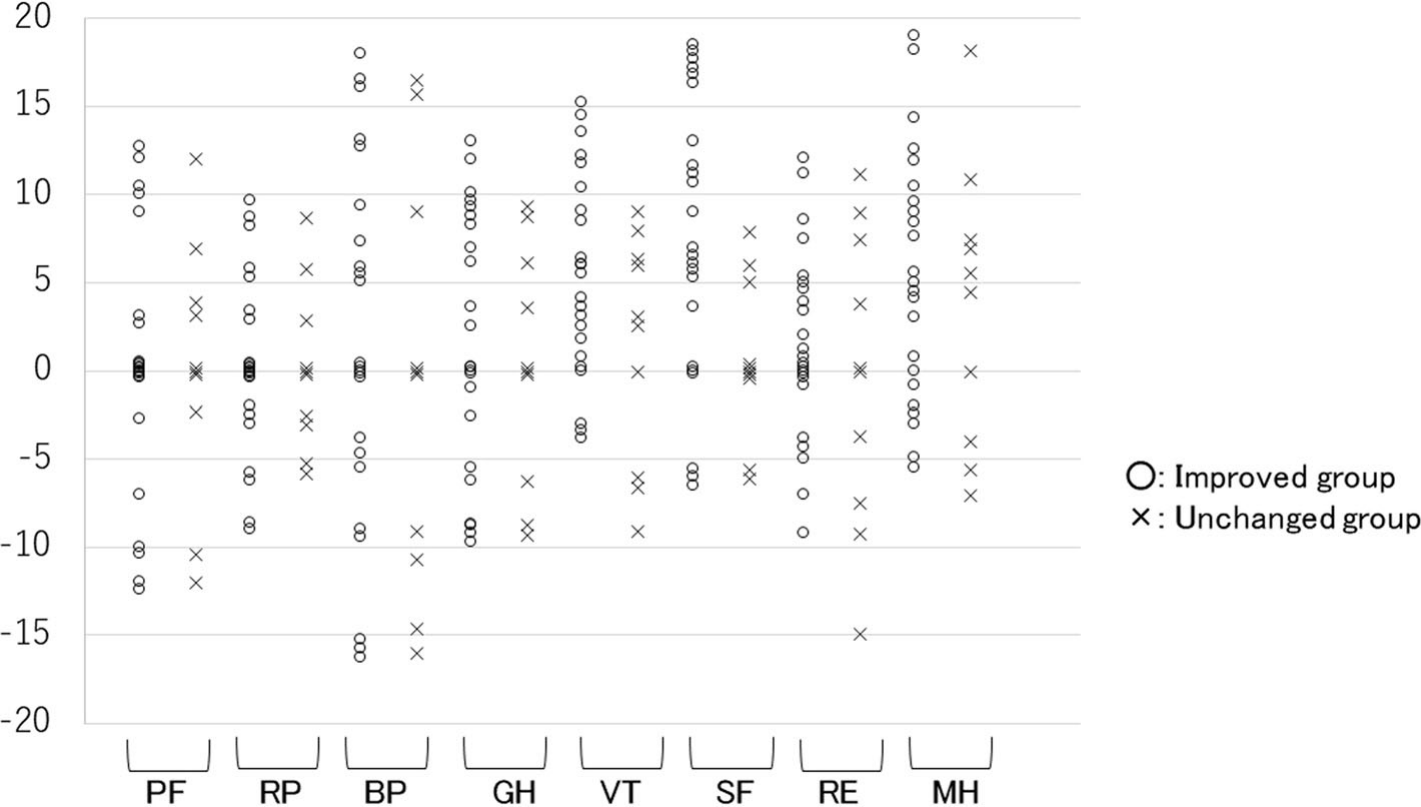
The distribution in the difference between the scale scores of each group before and after 3 months of treatment. PF, physical function; RP, role limitations because of physical health problems; BP, bodily pain; GH, general health perception; VT, vitality; SF, social function; RE, role limitations because of emotional problems; MH, mental health. The VT and SF score improvement rates in the improved group were 78% (18/23) and 74% (17/23), respectively, and only 3 patients had decreased scores on both scales

**Fig. 4:**
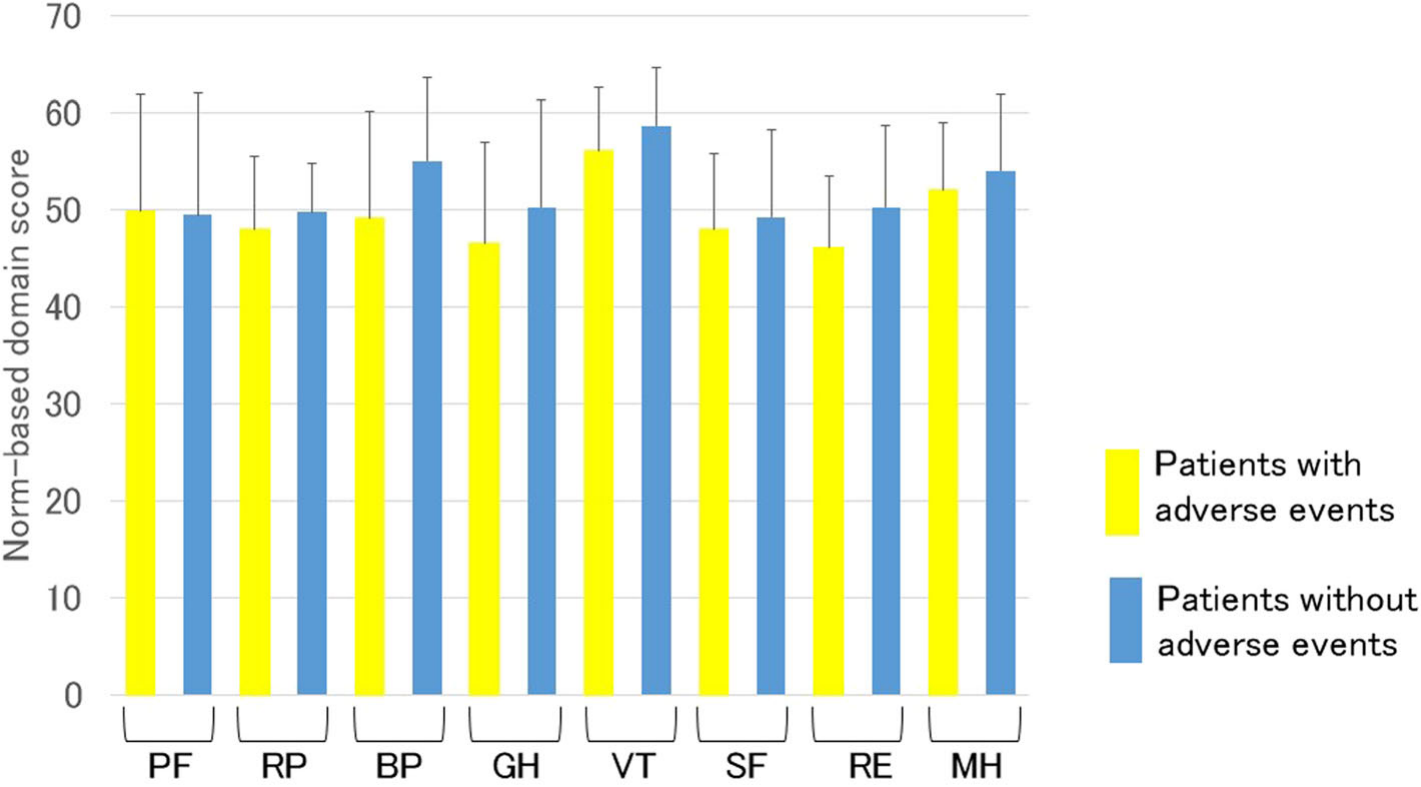
The comparison of the scale scores on the Medical Outcomes Study 36-Item Short Form 3 months after the initiation of sirolimus gel treatment for facial angiofibroma associated with tuberous sclerosis complex between patients with and without treatment-related adverse events. PF, physical function; RP, role limitations because of physical health problems; BP, bodily pain; GH, general health perception; VT, vitality; SF, social function; RE, role limitations because of emotional problems; MH, mental health. There were no significant differences in any scale scores between these two groups

We then assessed the influences of the therapeutic efficacy and adverse events of sirolimus gel treatment on the health-related QOL. Figure 2 shows a comparison of the scale scores of the SF-36 3 months after the initiation of sirolimus gel treatment for the improved group and the unchanged group. There were significant differences in two scale scores (VT and SF) between these two groups. Figure 3 shows the distribution in the difference between the scale scores of each group before and after 3 months of the treatment. The VT and SF score improvement rates in the improved group were 78% (18/23) and 74% (17/23), respectively, and only 3 patients had decreased scores on both scales. Figure 4 shows that there were no significant differences in any scale scores of the SF-36 between patients with and without adverse events after 3 months of sirolimus gel treatment.

## Discussion

TSC induces the development of various hamartomas throughout the body, including angiomyolipomas and angiofibromas. The mTOR signaling pathway has been reported to be activated in these tumors [1]. Sirolimus gel, an mTOR inhibitor, is effective against FA associated with TSC. In the UK management guidelines, topical mTOR inhibitors are recommended as the first line treatment for skin lesions associated with TSC [1].

To date, several studies have reported the influence of topical mTOR inhibitors treatment on patients’ QOL. Wataya-Kaneda et al. assessed the QOL improvement of patients with sirolimus gel treatment for FA associated with TSC using the Dermatology Life Quality Index (DLQI) and Children’s DLQI (CDLQI). They reported that the total scores on the DLQI and CDLQI after 12 weeks of treatment did not differ markedly between the sirolimus gel group and the placebo group [6]. Similar findings were reported in previous studies analyzing the QOL improvement of patients with topical mTOR inhibitors for FA associated with TSC using the DLQI and CDLQI [10, 11]. The DLQI and CDLQI are often used for QOL assessments in the treatment of skin diseases [6, 12, 13]. They are simple and comprised of 10 questions evaluating the disease impact on key aspects of patients’ lives [12–14]. However, there are few questions related to vitality and mental health, so the DLQI and CDLQI are not able to evaluate the mental health of patients sufficiently.

In this study, we used the SF-36 health survey to prospectively assess the changes in the physical and mental health of Japanese patients who had FA associated with TSC and were treated with sirolimus gel. The three scale scores associated with mental health, VT, SF, and MH, were significantly improved compared to before the treatment.

FAs influence the mental status of TSC patients and their families [11]. Since FAs often cause disfigurement, patients personality can become introverted, with reduced sociability [11]. Therefore, the treatment of FA might improve not only skin lesions but also mental health.

We further assessed the treatment efficacy and adverse events. It has been recognized that these two major factors are directly associated with patient satisfaction and health-related QOL during treatment. Patients in whom FA improved had significantly higher mental health scale scores for VT and SF than those whose condition did not improve. Furthermore, there was no marked influence of adverse events related to sirolimus gel treatment on the health-related QOL. These findings suggested that mental health became better in patients whose FA improved after administration of sirolimus gel. In this study, the adverse events with sirolimus gel treatment were tolerable, with only low-grade severity.

However, our study had several limitations. First, several surveys for the evaluation of health-related QOL have been developed, including as the Sickness Impact Profile (SIP) and the Functional Assessment of Cancer Therapy-General (FACT-G) [15, 16]. In the present study, we evaluated the health-related QOL using only the SF-36. However, the SF-36 questionnaire is not designed to assess the pediatric health-related QOL. As a result, we excluded children from this study. Amin et al. reported that topical sirolimus treatment for FA associated with TSC was most beneficial when started in childhood [17]. Further studies are required using other surveys for the evaluation of health-related QOL. Second, this study did not monitor patients over a long period. The long-term safety and tolerability of sirolimus gel treatment have not been sufficiently investigated. Thus, further studies on the influence of long-term sirolimus gel treatment upon health-related QOL are required.

## Conclusions

We evaluated the changes in the health-related QOL in patients with sirolimus gel treatment for FA associated with TSC by using the SF-36. Three scale scores associated with mental health, VT, SF, and MH, were significantly improved compared to before the treatment. Furthermore, improvements of the VT and SF scores were observed in the improved group compared with the unchanged group. However, further investigations are necessary to achieve a better understanding of the efficacy and safety of sirolimus gel treatment for FA associated with TSC.

## Data Availability

Data are available on reasonable request.

## Abbreviations

QOL: Quality of life
FA: Facial angiofibroma
TSC: Tuberous sclerosis complex
SF-36: Medical Outcomes Study 36-Item Short Form
mTORC1: mammalian target of rapamycin complex 1
ITSCCC: International Tuberous Sclerosis Complex Consensus Conference
PF: Physical function
RP: Role limitations because of physical health problems
BP: Bodily pain
GH: General health perception
VT: Vitality
SF: Social function
RE: Role limitations because of emotional problems
MH: Mental health
DLQI: Dermatology Life Quality Index
CDLQI: Children’s DLQI
SIP: Sickness Impact Profile
FACT-G: Functional Assessment of Cancer Therapy-General
